# Correlation Between Cannabidiol-Induced Reduction of Infarct Volume and Inflammatory Factors Expression in Ischemic Stroke Model

**DOI:** 10.18869/nirp.bcn.8.2.139

**Published:** 2017

**Authors:** Sepideh Khaksar, Mohammad Reza Bigdeli

**Affiliations:** 1. Department of Physiology, Faculty of Biological Sciences and Technology, Shahid Beheshti University, Tehran, Iran.

**Keywords:** Cannabidiol, Cerebral ischemia, Infarction, TNFR1, NF- κB

## Abstract

**Introduction:**

Recent studies demonstrated that cannabidiol had neuroprotective property. There is some evidence about effective role of cannabidiol in reduction of ischemic damages. It has been reported that infarct size is influenced by various factors after MCAO, including inflammatory factors. The aim of the present study was to evaluate the effect of cannabidiol on infarction volume and correlation of infarct size with tumor necrosis factor receptor 1 (TNFR1), and nuclear factor kappa-light-chain-enhancer of activated B cells (NF-κB) expression.

**Methods:**

Using stereotaxic surgery, guide cannula was implanted in the right lateral ventricle. Cannabidiol (50, 100, and 200 ng/rat) was injected through ntracerebroventricular (i.c.v.) route for 5 consecutive days . Then, the rats underwent 60 minutes of right middle cerebral artery occlusion (MCAO). After 24 h reperfusion, the infarct volume in total, cortex, piriform cortex-amygdala (Pir-Amy), and striatum areas of hemisphere were assessed. The expression of inflammatory factors such as TNFR1 and NF-κB in these regions were also studied.

**Results:**

The present results indicate that in the MCAO-induced cerebral ischemia, administration of cannabidiol (100 and 200 ng/rat) causes a significant reduction in infarction volume in comparison with the vehicle group. Also, there were significant correlations between decrease of regional infarct volume and TNFR1/NF-κB expression.

**Conclusion:**

The results of this study indicate that cannabidiol reduced cerebral infarction possibly through diminishing TNFR1/NF-κB-induced neurotoxicity in transient focal cerebral ischemia.

## 1. Introduction

Inflammation has been recognized as a key contributor to the pathophysiology of cerebral ischemia and other forms of ischemic brain injury (Lakhan, Kirchgessner, & Hofer, 2009). There is growing evidence that this event is involved in all stages of the ischemic cascade, from the early intravascular events after arterial occlusion to the late regenerative alterations leading to brain damage and tissue repair (Iadecola & Anrather, 2011). Inflammatory process in cerebral ischemia is characterized by rapid activation of microglia, production of pro-inflammatory mediators, and infiltration of various types of inflammatory cells (including neutrophils, monocyte/macrophages, and other cells) into the ischemic brain tissue, which collaboratively contribute to ischemic brain injury (Jin, Yang, & Li, 2010).

Immediately after interruption of blood supply, pro-inflammatory mediators like cytokines and chemokines are excreted. By progression of ischemic process, cell death creates a new phase of inflammatory response. Ischemic cells activates toll-like receptor 4 (TLR-4) that in turn initiates pathways linked to the transcription of many pro-inflammatory gene encoding cytokines such as interleukin-1β (IL-1β) and tumor necrosis factor-α (TNF-α). Thus, modulation of inflammatory processes during cerebral ischemia might protect ischemic brain tissue (Iadecola & Anrather, 2011). It has been demonstrated that inhibiting of inflammatory response decreases infarct size and improves neurological deficit in experimental ischemic stroke (Wang, 2005). Stroke is the most frequent cause of permanent disability. Strokes can be categorized into ischemic and hemorrhagic.

Ischemic strokes are due to interruption of the blood supply to the brain, while hemorrhagic stroke results from the rupture of a blood vessel rupture or an abnormal vascular structure (Bigdeli, 2011). About 87% of strokes are ischemic (Donnan, Fisher, Macleod, & Davis, 2008). Intriguingly, brain ischemia has been linked to a variety of diseases or abnormalities in individuals which are vulnerable to cerebral ischemia, including sickle cell anemia, compressed blood vessels, aneurysm, and patients with a history to heart attack. Thus, prevention and induction of ischemic tolerance are the main aims of researchers and neurologists. On the other hand, cannabidiol (CBD), a non-psychotropic natural cannabinoid, is generally found in relatively high concentrations in cannabis plant. The past several years, interest in CBD has increased considerably, and represents one of the most promising candidates for present research because of the discovery of its remarkable anti-oxidative, anti-inflammatory, and neuroprotective properties (Esposito et al., 2009; Hampson, Grimaldi, Axelrod, & Wink, 1998; Iuvone et al., 2004), actions that occur for the most part independent of the cannabinoid CB1 and CB2 receptors (De Petrocellis & Di Marzo, 2010).

Thus, cannabidiol can influence the main signaling pathways leading to ischemic damages. It was reported that CBD had the ability to inhibit the inflammation in cerebral ischemia (Castillo, Tolon, Fernandez-Ruiz, Romero, & Martinez-Orgado, 2010; Pazos et al., 2012).

Probably, the neuroprotection exerted by cannabidiol is partly due to their anti-inflammatory potential. The purpose of the present study was to assess the effects of pre-treated cannabidiol on cerebral infarction and TNFR1/NF-κB expression during ischemic injury in the rat model of transient focal cerebral ischemia to determine whether size of infarction is correlated to NF-κB and TNFR1 expression in cannabidiol received groups.

## 2. Methods

### 2.1. Animals and group assignment

Adult male Wistar rats (250–350 g) were caged under controlled temperature (22±2°C) and constant humidity, with 12 h light/dark cycle. All experimental animals were treated based on National Institute of Health and Guide for the care and use of laboratory animals (NIH Publications No. 80-23) revised in 1996. The study was approved by the Ethics Committee of the Shahid Beheshti university of Iran. We tried our best to minimize the number of study animals. Rats were divided randomly into 5 main groups (control, vehicle, 50, 100, and 200 ng/rat of cannabidiol) of 80 animals and sham group with 10 animals.

Each main group was subdivided to ischemia-operated (n=6) for evaluation of infarct volume and intact (n=10) subgroups. The intact subgroup was subdivided for assessment of NF-κB and TNFR1 expression in total (n=5), cortex, piriform cortex-amygdala (Pir-Amy), and striatum (n=5) areas of hemisphere. Furthermore, sham-operated group (n=10) was defined for expression of NF-κB/ TNFR1 evaluation separately. Apart from control, sham, and vehicle received groups; treatment groups received cannabidiol (50, 100 and 200 ng/rat) for 5 consecutive days through intracerebroventricular (ICV) injection 7 days after stereotaxic surgery. Ischemia-operated subgroups were subjected to 60 min of middle cerebral artery occlusion (MCAO). Twenty-four hours later, infarct volume measurement was performed separately. In the control group, all steps were similar to stereotaxic/ ischemia-operated group without receiving any treatment. Sham-operated animals underwent the same surgery procedure, except for recieving the filament. For western blot technique in sham-operated and intact subgroup, all steps were similar to main groups without any surgery procedure.

### 2.2. Drug administration

Cannabidiol (Tocris, UK) was dissolved in a mixture of dimethylsulfoxide (DMSO; 10%) and phosphate buffer solution (PBS, 90%) (Shirazi-zand, Ahmad-Molaei, Motamedi, & Naderi, 2013). Microinjection of cannabidiol into right lateral ventricle was in a volume of 2 μL. ICV injection of cannabidiol at doses of 50, 100, and 200 ng/rat (Shirazi-zand et al., 2013) was given by inserting a 30-gauge injection cannula 1 mm beyond the tip of the guide cannula to the site of injection. The injection cannula was connected with a polyethylene tube to a Hamilton syringe. Injection (1μL) was done over 60s. As, cannabidiol was injected in the treatment groups, the vehicle group received DMSO 10% through ICV injection. Analysis between control and vehicle groups was done because there are evidences about possible neuroprotective role of vehicle (dimethylsulfoxide) (Di Giorgio et al., 2008).

### 2.3. Stereotaxic surgery

Animals were anesthetized and put in a stereotaxic frame (Stoelting Instruments, USA) with flat skull position. After a midline incision, the skin and underlying periosteum were retracted. Steel guide cannula (23 gauge) was placed 1 mm above the right lateral ventricle according to stereo-taxic coordinates: AP, −0.58 mm posterior to the bregma; L,±1.4 mm from midline; and V, −1.6 mm relative to skull (Paxinos, Watson, & Emson, 1980). The cannula was anchored to the skull with dental cement. Animals were then placed in animal house for 7 days, after which stereotaxic-operated. Injection of 2 μL of methylene blue solution into the lateral ventricle wascarried out to verify nicroinjection site. After decapitation, the brain was removed and fixed in 10% formalin solution for 48 h. Sections were checked by macroscop to verify the correct placement of cannula in the lateral cerebral ventricle.

### 2.4. Focal cerebral ischemia

On the fifth day, rats received cannabidiol 30 min prior to MCAO surgery. In this surgery, the middle cerebral artery (MCA) was sutured by the intraluminal technique described by Longa et al. (Longa, Weinstein, Carlson, & Cummins, 1989). Briefly, the left carotid region was exposed and the external carotid and common carotid arteries were ligated with a suture. A 3-0 coated monofilament nylon suture (Nylon, home-made), whose tip had been rounded by heating and coated with poly-l-lysine (Sigma, USA) was inserted from the carotid bifurcation into the internal carotid artery until a mild resistance was felt (18–19 mm), thereby blocking the origin of the MCA and thus blood flow. Reperfusion was established by suture removal after 60 min of ischemia. Rectal temperature was monitored (Citizen-513w, CITIZEN, United Arab Emirates) and maintained at 37°C by surface heating and cooling during surgery.

### 2.5. Infarct volume

On the fifth day, rats received cannabidiol 30 min prior to MCAO surgery. In this surgery, the middle cerebral artery (MCA) was sutured by the intraluminal technique described by Longa et al. (Longa et al., 1989). Briefly, the left carotid region was exposed and the external carotid and common carotid arteries were ligated with a suture. A 3-0 coated monofilament nylon suture (Nylon, home-made), whose tip had been rounded by heating and coated with poly-l-lysine (Sigma, USA) was inserted from the carotid bifurcation into the internal carotid artery until a mild resistance was felt (18–19 mm), thereby blocking the origin of the MCA and thus blood flow. Reperfusion was established by suture removal after 60 min of ischemia. Rectal temperature was monitored (Citizen-513w, CITIZEN, United Arab Emirates) and maintained at 37°C by surface heating and cooling during surgery.

### 2.6. Western blot analysis

The expression of NF-κB and TNFR1 protein was assessed by Western blotting following sample extraction and SDS-PAGE. Assessment of NF-κB and TNFR1 expression has been carried out in total, core, and penumbra areas of hemisphere separately. Tissue samples were homogenized in lysis buffer at 4°C for 1 min and the extract was centrifuged at 12,000 rpm at 4°C for 20 min. The supernatant was treated like the tissue extract. SDS sample buffer was added to aliquots of tissue extracts. Then, samples were heated at 100°C for 5 min. Protein was separated by SDS-PAGE (10% gel). Blotting was done by semi-dry type blotting (BIORAD). The blots were blocked with 2% non-fat dry milk in Tris buffer saline in 0.1% tween 20 at 4°C for 75 min. They were incubated for 18 h with rabbit anti-NF-κB polyclonal (1:500 dilution), goat anti-TNFR1 polyclonal antibodies (1:500 dilution) and rabbit anti-β-actin (1:1000 dilution) in TBS-T, followed by goat anti-rabbit and rabbit anti-goat secondary antibodies (1:500 dilution) in TBS-T for 90 min separately. NF-κB and TNFR1 immune-reactive proteins were detected with advanced chemiluminescence (Enhanced Chemiluminescence, Amersham Biosciences) and film exposure. Anti-NF-κB, anti-TNFR1, goat anti-rabbit and rabbit anti-goat antibodies were purchased from Santa Cruz. The signal intensity of the blots was measured by an image analysis system (Image j, version 1.46 r).

### 2.7. Statistical analysis

Data of infarct volume, NF-κB, and TNFR1 expression were analyzed by using two-way and one-way ANOVA analysis of variance, respectively (SPSS v.22.0 post hoc LSD). Correlation analyses was done by Pearson’s linear regression. Data were expressed as mean±SEM and value of P<0.05 was considered significant.

## 3. Results

**Figure 1.**
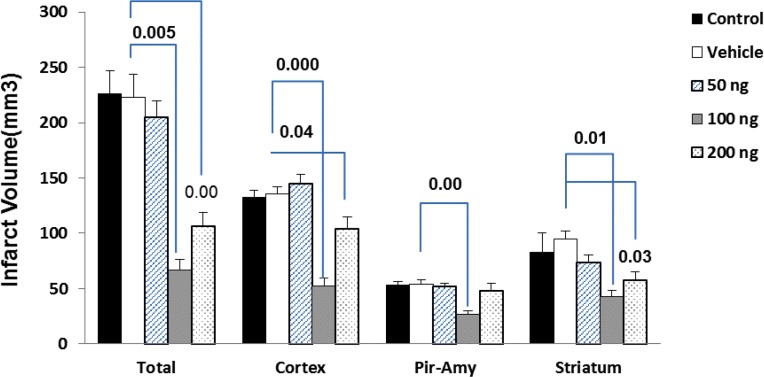
Effect of cannabidiol at doses of 50, 100, and 200 ng on MCAO-induced infarct volume in total, cortex, piriform cortex-amygdala (Pir-Amy), and striatum. Infarction volume was significantly reduced at dose of 100 and 200 ng of cannabidiol in comparison with vehicle group. Values are expressed as the mean±SEM (n=6). P<0.00 compared with vehicle-treated group.

The effect of cannabidiol-induced protection on infarct volume is as follows: The total infarct volume in the vehicle group was 223.18±11.1 mm3. Administration of cannabidiol at doses of 100 ng/rat (67.21±7.1 mm3) and 200 ng/rat(105.04±8.2 mm3) resulted in a reduction of infarct volume significantly (P=0.005 and P=0.00, respectively). Moreover, the infarct size has not significantly changed in animals with CBD at a dose of 50 ng/rat (202.18±14 mm3) compared to the vehicle group. Furthermore, CBD in doses of 100 (51.49±12.5 mm3, P<0.001) and 200 ng/rat (102.32±19.19 mm3, P=0.04) significantly decreased brain infarct size in the cortex area compared with vehicle group (132.5±11.2 mm3). Shrinkage in infarct volume exerted by two doses of CBD (100 and 200 ng) was also seen in the striatum (P<0.05). In piriform cortex-amygdala (Pir-Amy) area only dose of 100 ng/rat CBD could decrease infarction (P=0.00). Comparison between control and vehicle groups was not significant (Figure 1).

**Figure 2.**
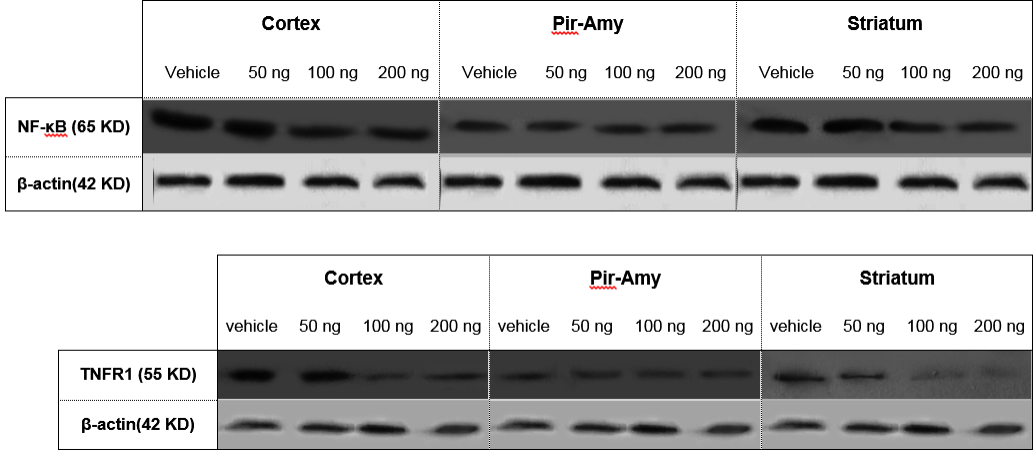
Western blot of NF-κB and TNFR1 proteins in cortex, piriform cortex-amygdala (Pir-Amy), and striatum of vehicle, received of 50, 100, and 200 ng cannabidiol groups. Analysis of protein bands after normalization with β-action as loading control has been carried out. The NF-κB and TNFR1 expression was decreased at doses of 100 and 200 ng cannabidiol in cortex and striatum, compared to the vehicle group.

Correlation between infarct volume and NF-kB and TNFR1 expression is as follows: Using of western blot technique showed that TNFR1 and NF-κB is expressed in total of hemisphere, cortex, piriform cortex-amygdala (Pir-Amy), and striatum areas in rat brain. Present study indicated that TNFR1 and NF-kB expression in total of hemisphere, cortex, and striatum were reduced at dose 100 and 200 ng/rat of CBD-received groups in compared with vehicle group. Comparison between control, sham and vehicle groups was not significant (data not shown) (Figure 2).

### 3.1. Total of cerebral hemisphere

**Figure 3.**
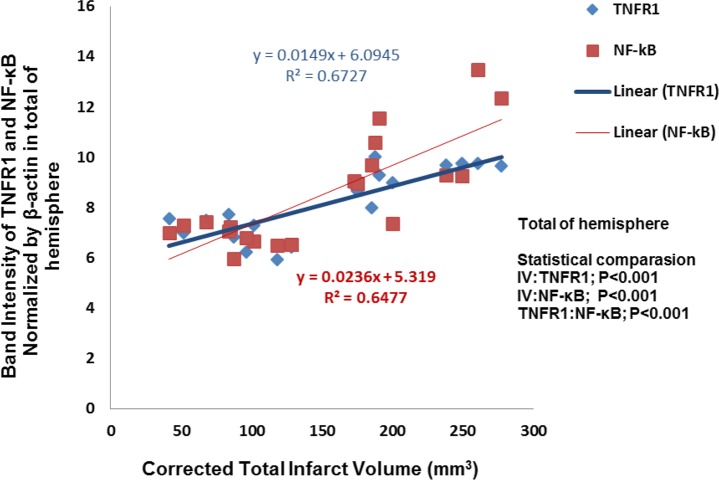
Correlation between infarction volume and TNFR1/NF-kB expression in total of hemisphere rat brain. There is significant correlation between cannabidiol-induced decrease of the infarct volume and reduction of TNFR1/NF-kB expression (P<0.001, P<0.001, respectively) (n=5).

Correlation between decrease of the total infarct volume and TNFR1 expression in cerebral hemisphere exerted by cannabidiol was significant (P<0.001, R^2^=0.6727). The significant correlation between decrease of the total infarct volume and NF-κB expression has also been observed (P<0.001, R^2^=0.6477) (Figure 3).

### 3.2. Cortex

**Figure 4.**
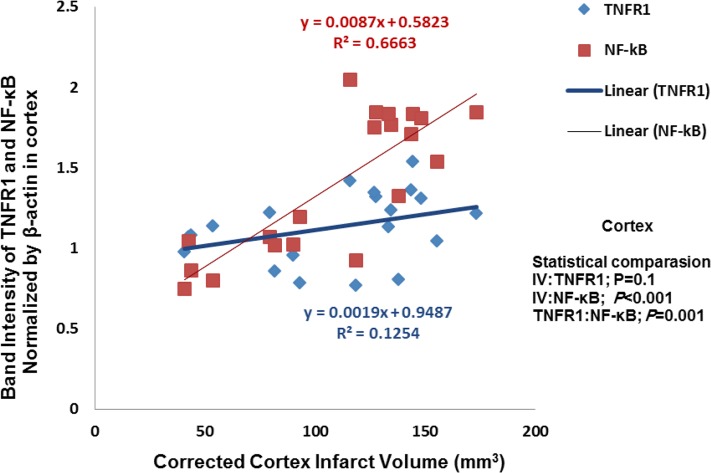
Correlation between infarction volume and TNFR1/NF-kB expression in cortex. Correlation between reduction of infarct volume and NF-kB expression exerted by cannabidiol was significant (P<0.001) (n=5).

Correlation analysis between cannabidiol-treated infarct volume and inflammatory factors in cortex was carried out. Result show that decrease of the infarct volume induced by cannabidiol was significantly correlated with reduction of NF-κB expression (P<0.001, R^2^=0.6663), whereas this correlation between infarct volume and TNFR1 was not significants (Figure 4).

### 3.3. Piriform cortex-amygdala (Pir-Amy)

**Figure 5.**
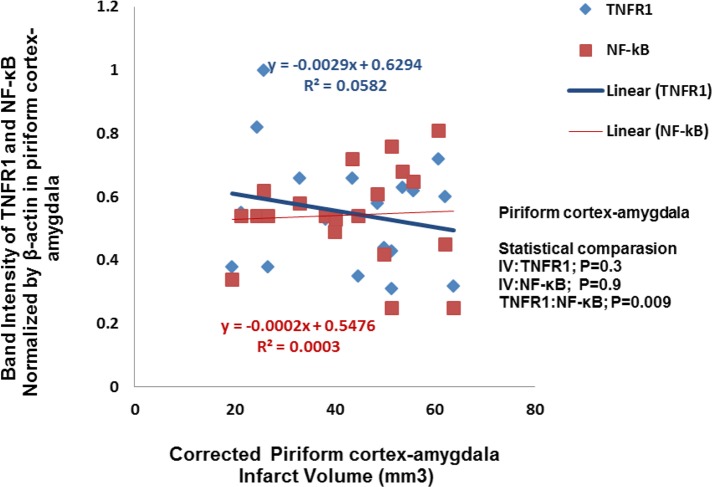
Correlation between infarction volume and TNFR1/NF-kB expression in piriform cortex-amygdala. This correlation statistically was not significant (n=5).

The correlation decrease of infarct volume with TNFR1 and NF-κB expression statistically was not significant (Figure 5).

### 3.4. Striatum

**Figure 6.**
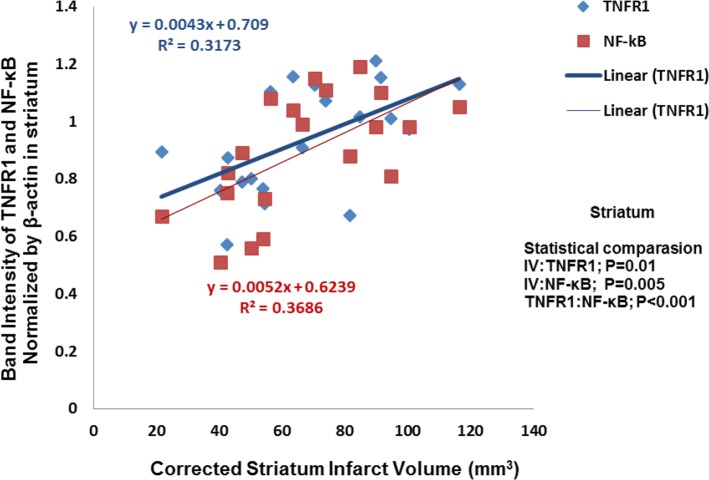
A significant correlation was found between infarction volume and TNFR1/NF-kB expression in striatum. There is significant correlation between cannabidiol-induced decrease of the infarct volume and TNFR1/NF-kB expression, respectively (P=0.01, P=0.005, respectively) (n=5).

There is a significant correlation between cannabidiol-induced reduction of the infarct volume and TNFR1/NF-κB expression in striatum (P=0.01, R^ 2 ^=0.3173; P=0.005, R^ 2 ^=0.3686) (Figure 6).

## 4. Discussion

The present work indicates that ICV administered cannabidiol (100 and 200 ng) in animal model of cerebral ischemia reduced infarct volume (total) significantly. The protective effect of CBD at dose of 100 ng/rat on infarction has also been observed in cortex, piriform cortex-amygdala (Pir-Amy), and striatum areas. Also, cortical and striatal infarct volume decreased at dose of 200 ng/rat CBD significantly. These data are supported by other experimental studies showing that cannabidiol may attenuate infarction (Braida et al., 2003; Mishima et al., 2005; Pazos et al., 2013). This route of administration has been done for the first time in animal model of cerebral ischemia. The previous investigations have mostly been designed on therapeutic approach, while induction of ischemic tolerance is innovation of this research. Notably, the difference between present research and others relates to the main purpose of study, the route of administration, model of ischemic induction, and duration of occlusion. Cannabidiol exerted a neuroprotective effect with a dose-dependent bell-shaped curve as other studies reported (Braida et al., 2003; Mishima et al., 2005). Cannabidiol at lower doses (50 ng) could not prevent the infarct volume induced by the severe condition, whereas 100 ng/rat may be introduced as optimal dose and the CBD 200 ng/rat dose affected infarction lower than 100 ng. We suppose that higher dose of CBD 200 ng/rat will enlargethe infarct volume induced by MCA occlusion. The dose-response curve of cannabidiol should be examined in details. Infarct volume is a common index to determine the extent of ischemic brain injury following focal cerebral ischemia. Focal cerebral ischemia has been created in animals to simulate human ischemic stroke. Cerebral ischemia results in necrosis in the ischemic core and apoptosis in adjacent area (penumbra), leading to well-demarcated infarction (Lin, He, Wu, Khan, & Hsu, 1993).

Moreover, the results of the present study demonstrate that there are significant correlations between infarct volume (total) and NF-κB and TNFR1 expression (total). Reduction of infarction by cannabidiol is correlated with down-regulation of NF-κB and TNFR1 expression. This relationship between the infarct size and NF-κB and TNFR1 expression has also been observed in cortex and striatum areas. TNF-α factor is an important trigger of inflammatory cascade. It can perform a pro-inflammatory/pro-apoptotic role. TNF-α signal acts through two TNF-α receptors; TNFR1 and TNFR2. TNFR1 has the most important role in the biological activity of TNF-α and is expressed in the most tissues, while TNFR2 is expressed in immune cells.

TNF-α binding to TNFR1 will trigger a series of intracellular events that ultimately activate the transcription factor NF-қB. NF-қB increases the expression of a group of genes that enhances inflammation (Wajant, Pfizenmaier, & Scheurich, 2003). Furthermore, sustained presence of TNF-α activates caspase 8 that leads to apoptosis. TNF-induced cell death plays only a minor role compared to its overwhelming functions in the inflammatory process (Gaur & Aggarwal, 2003). The involvement and upregulation of TNF-α and TNFR in many pathological manifestations including cerebral ischemia have been implicated (Botchkina, Meistrell, Botchkina, & Tracey, 1997). The detrimental effects of TNF-α in cerebral ischemia have been attributed to a number of mechanisms, namely increasing the blood brain barrier permeability (Megyeri et al., 1992), stimulating the production of matrix-degrading metalloproteinase (Rosenberg, Dencoff, Correa, Reiners, & Ford, 1996), increasing leukocyte adhesion to brain vessels (Robbins et al., 1987), and direct toxic effects on brain capillaries (Goldblum & Sun, 1990). It has been reported that inhibiting TNF-α signaling by pharmacologic agents reduced infarct volume in preclinical model (Hosomi et al., 2005; Nawashiro, Martin, & Hallenbeck, 1997). NF-κB (a redox-sensitive transcription factor) is a part of intracellular pathway regulating the transcription of hundreds of genes involved in cell survival and inflammation. There is much evidence that NF-κB is activated in cerebral ischemia and may contribute to infarction in permanent MCAO-operated mice (Nurmi, 2004).

Despite its well-known role as an anti-apoptotic factor, NF-κB contributes to neuronal cell death in cerebral ischemia. Activation of cell death pathways such as NF-κB and impairment of pro-survival signaling pathways are triggered by hypoxia, free radicals, and inflammatory mediators (Interleukin-1β and Interleukin-6) in cerebral ischemia (Ridder & Schwaninger, 2009). The NF-κB p65/p50 protein is present as a latent, inactive, inhibitor protein (IκB)-bound complex in the cytoplasm that the active p65 NF-κB is released by increased phosphorylation of IκB-α. The active p65 NF-κB subsequently is translocated to the nucleus to induce various inflammatory (including IL-1 and IL-6) and apoptotic gens expression (Rothwarf & Karin, 1999). Therefore, we assume that reduction of infarct volume by CBD might be mediated through inhibition of TNFR1 and NF-κB.

Reduction of TNFR1 and NF-κB expression is attributed to anti-inflammatory property of cannabidiol. CBD exerts immunosuppressive action by decreased TNFα production after treatment with lipopolysaccharide (Ruiz-Valdepenas et al., 2011). In addition, CBD reduced elevated–TNF-α level in experimental diabetes (El-Remessy et al., 2006). CBD provoked a reduction in TNF-α release and NF-κB expression in the animal model of Alzheimer disease (Esposito et al., 2011). In accordance with this data, cannabidiol has exerted its anti-inflammatory effect through TNF-α inhibition in cerebral ischemia (Castillo et al., 2010; Lafuente et al., 2011; Pazos et al., 2012). It has been reported that an increase in inducible nitric oxide synthase (iNOS) expression was prevented by CBD probably through inhibition of P38 mitogen-activated protein kinases (MAPK p38) and NF-κB activation (Esposito et al., 2006). As mentioned before, TNF-α binding to TNFR1 can trigger apoptosis pathway through caspase 8 and TNFR1 as activator of NF-κB promotes apoptosis. Therefore, the direct relationship may exist between TNFR1/ NF-κB and neuronal death, which is defined as infarction in cerebral ischemia. In an ischemic brain, leukocytes and activated microglia produce various pro-inflammatory molecules like iNOS, matrix metalloproteinases (MMPs), IL-1β and TNF-α, and continue to excrete free radicals. These activities give rise to secondary brain injury, increased inflammation, disruption of BBB, brain edema, and ultimately cell death (Tobin et al., 2014). Due to these circumstances, inflammation is an initiator of cell death in the delayed phase of ischemic injury. Thus, suppression of inflammation by protective agents will attenuate severity of ischemic injury.

In conclusion, the present study has demonstrated pre-treatment of cannabidiol ameliorated infarction and ischemic injury through suppression of TNFR1 ad NF-κB expression. Moreover, present investigation indicates that sizes of infarction in the cerebral hemisphere, cerebral cortex, and striatum are highly correlated with TNFR1 ad NF-κB expression.
